# Ascorbic Acid-Induced Photosynthetic Adaptability of Processing Tomatoes to Salt Stress Probed by Fast OJIP Fluorescence Rise

**DOI:** 10.3389/fpls.2021.594400

**Published:** 2021-08-16

**Authors:** Xianjun Chen, Yan Zhou, Yundan Cong, Pusheng Zhu, Jiayi Xing, Jinxia Cui, Wei Xu, Qinghua Shi, Ming Diao, Hui-ying Liu

**Affiliations:** ^1^Department of Horticulture, Agricultural College, Shihezi University, Shihezi, China; ^2^Key Laboratory of Special Fruits and Vegetables Cultivation Physiology and Germplasm Resources Utilization of Xinjiang Production and Construction Corps, Shihezi, China; ^3^Department of Biological Science, Life Science and Technology School, Lingnan Normal University, Zhanjiang, China; ^4^Department of Vegetables, Science and Engineering, Shandong Agricultural University, Tai'an, China

**Keywords:** processing tomatoes, ascorbic acid, NaCl stress, fast OJIP fluorescence rise curve, photosynthesis

## Abstract

In this study, the protective role of exogenous ascorbic acid (AsA) on salt-induced inhibition of photosynthesis in the seedlings of processing tomatoes under salt stress has been investigated. Plants under salt stress (NaCl, 100 mmol/L) were foliar-sprayed with AsA (0.5 mmol/L), lycorine (LYC, 0.25 mmol/L, an inhibitor of key AsA synthesis enzyme l-galactono-γ-lactone dehydrogenase activity), or AsA plus LYC. The effects of AsA on fast OJIP fluorescence rise curve and JIP parameters were then examined. Our results demonstrated that applying exogenous AsA significantly changed the composition of O-J-I-P fluorescence transients in plants subjected to salt stress both with and without LYC. An increase in basal fluorescence (*F*_o_) and a decrease in maximum fluorescence (*F*_m_) were observed. Lower K- and L-bands and higher I-band were detected on the OJIP transient curves compared, respectively, with salt-stressed plants with and without LYC. AsA application also significantly increased the values of normalized total complementary area (S_m_), relative variable fluorescence intensity at the I-step (V_I_), absorbed light energy (ABS/CS_m_), excitation energy (TR_o_/CS_m_), and reduction energy entering the electron transfer chain beyond Q_A_ (ET_o_/CS_m_) per reaction centre (RC) and electron transport flux per active RC (ET_o_/RC), while decreasing some others like the approximated initial slope of the fluorescence transient (M_o_), relative variable fluorescence intensity at the K-step (V_K_), average absorption (ABS/RC), trapping (TR_o_/RC), heat dissipation (DI_o_/RC) per active RC, and heat dissipation per active RC (DI_o_/CS_m_) in the presence or absence of LYC. These results suggested that exogenous AsA counteracted salt-induced photoinhibition mainly by modulating the endogenous AsA level and redox state in the chloroplast to promote chlorophyll synthesis and alleviate the damage of oxidative stress to photosynthetic apparatus. AsA can also raise the efficiency of light utilization as well as excitation energy dissipation within the photosystem II (PSII) antennae, thus increasing the stability of PSII and promoting the movement of electrons among PS1 and PSII in tomato seedling leaves subjected to salt stress.

## Introduction

Xinjiang Province is one of the main producing regions of processing tomatoes in China. However, because of excessive use of fertilizers, unreasonable methods of irrigation and drainage, and the special climatic conditions (such as dryness due to less rain and strong evaporation in Xinjiang), the area is threatened by soil salinization, and soil secondary salinization has exceeded more than half of the total cultivated land dedicated to processing tomatoes. In recent years, this salinization problem became more and more serious, as it results in declines in photosynthetic rates, environmental resistance, and yield and quality of processing tomatoes. Increasing levels of salt in soil have become a primary abiotic stress that limits the productivity of processing tomatoes in Xinjiang Province (Pang et al., 2005). High salinity inhibits crop growth by disrupting a range of physiological processes involved in cell metabolism, especially photosynthesis (Thagela et al., 2017; Yang et al., 2020). Photosynthesis is the primary metabolic mechanism in plants (Singh et al., 2014) and is particularly sensitive to salinity (Ashraf and Harris, 2013). Salinity causes stomatal closure, destruction of chlorophyll pigments, inhibition of photochemical efficiency of photosystem II (PSII), and CO_2_ assimilation (Dietz and Pfannschmidt, 2011). The presence of salt increases the buildup of reactive oxygen species (ROS) (Oukarroum et al., 2015; Hossain et al., 2017). The accumulated ROS severely damage the ultrastructure of photosynthetic apparatus, disrupting the use of light energy and causing the photoinhibition of PSII, consequently leading to plant growth retardation, and even cell and plant death (Jiang et al., 2017). Therefore, it is very important for plants to increase their salt tolerance and maintain their economic yield to counteract the adverse effects of salt stress on photosynthesis.

Reduced ascorbic acid (AsA) is by far the most abundant low-molecular weight antioxidant in plants, and AsA plays a key role in protecting plants from ROS generated during normal metabolic processes and under stress conditions including wounding, pathogen attack, salt, heat, light, and ozone exposure (Conklin, 2010). As an efficient electron donor and important non-enzymatic antioxidant, AsA constitutes a redox buffer that keeps the intracellular environment reduced and maintains the cellular redox homeostasis by directly or indirectly scavenging ROS (including H_2_O_2_, ${\text{O}}_{2}^{-}$, ·OH^−^, and ^1^O_2_). AsA can act as a signaling agent participating in the interaction with the environment (Linster and Clarke, 2008). AsA is also associated with several biosynthetic pathways, such as the xanthophyll cycle, which guard against the negative effects of surplus energy from excitation. Furthermore, many authors have demonstrated in the past years that AsA still exerts other significant roles in a wide range of processes, such as plant growth control, α-tocopherol regeneration, cell division and expansion regulation, resistance to insect feeding (Foyer and Graham, 2011), carbon–nitrogen metabolism, fruit quality and seed germination, as well as the expression of genes related to hormone synthesis and signal transduction. The cellular AsA pool size in plants can be regulated by the coordinated action of many related enzymes, and an enhanced AsA level has been confirmed to increase the tolerance and adaptation of crops to abiotic stresses such as high light intensity, cold, drought, salinity, UV-B, and ozone stresses (Xie et al., 2009). Similarly, exogenous application of AsA to plants has been shown to confer tolerance to several abiotic stresses in many crop plants (Xie et al., 2009; Wang et al., 2014; Alhasnawi et al., 2016; Akram et al., 2017; Penella et al., 2017). Our previous research demonstrated that optimal concentrations of exogenous AsA could relieve the salt-induced inhibition on the seedling growth of processing tomatoes, increasing its adaptability to salt stress (Supplementary Materials). It also showed that tomato salt tolerance improvement induced by exogenous selenium, glutathione (GSH), and nitric oxide (NO) is accompanied by the increase of endogenous AsA content and AsA/dehydroascorbate (DHA) ratio (He et al., 2015; Wen et al., 2018; Zhou et al., 2018).

The mechanisms of salt tolerance are very complex. Recent studies aiming to understand the mechanisms of AsA effects on plant salt tolerance have shown that salt tolerance induced by endogenous AsA or application of exogenous AsA mainly ascribed to the improvement of antioxidant, ion balance, and osmotic balance regulation (Wang et al., 2019). Although the photosynthetic apparatus is the most sensitive component for evaluating the degree of salt stress damage, few studies concerning the protection mechanisms of exogenous AsA on its structure and function under salt stress were carried out and more detailed ones are needed. The fluorescence transient (OJIP) technique is a quick, precise, and non-invasive method for reliably detecting and analyzing the effects of abiotic and biotic stress on the composition and processes of PSII and photosystem I (PSI), such as the redox condition of both photosystems, alterations in the main photosynthetic processes, and the efficiency of the transfer of electrons via the cross-system bond to the electron acceptors on the PSI acceptor node (Brestic et al., 2014). The conditions observed in the JIP test as well as the composition of the OJIP transient are easily affected by various stressors and have also been successfully employed in assessing and screening the tolerance of plants to environmental stresses (Chen et al., 2016; Çiçek et al., 2017; Essemine et al., 2017; Banks, 2018; Maliba et al., 2019). As such, we created tomato plants with various levels of endogenous AsA by applying AsA, the leaves of tomato seedlings that have been exposed to salt. Hence, we constructed tomato plants with different endogenous AsA levels by spraying salt-stressed tomato seedling leaves with AsA, Lycorine (LYC, an inhibitor of key AsA synthesis enzyme l-galactono-γ-lactone dehydrogenase activity), or AsA and LYC. The purpose of this experiment was to use OJIP transient analyses to assess the processes by which AsA impacts the photosystem stability, the processes of the photosynthetic electron transport chain, and the relationship between energy use and expenditure in the photosynthetic system of processing tomatoes subjected to salt stress. Increasing the exogenous AsA to expand PSII stability and regulate the photosynthetic activity and efficiency of the distribution of energy significantly contributed to mitigating the negative effects of salt stress, namely, the inhibition of photosynthetic processes and plant growth.

## Materials and Methods

### Plant Materials and Treatments

Processing tomatoes (*Lycopersicon esculentum* L. cv. Ligeer87-5) were utilized for the relevant experiments, which were conducted hydroponically in a solar-powered greenhouse in Shihezi University, Xinjiang Uygur Autonomous Region, China. We transplanted seedlings (each with two euphylla) to a 12 L plastic container containing 10 L of full-strength, oxygenated Hoagland nutrient mixture (*pH* = 6.2).

Five treatments were performed on the seedlings of processing tomatoes after allowing them to pre-culture for 7 days. The NaCl was added to the nutrient mixtures, and LYC and AsA were sprayed on the leaves. This was made for five different treatments: (1) no added NaCl, no sprayed AsA and LYC (Control); (2) 100 mmol/L NaCl (NaCl); (3) 100 mmol/L NaCl and 0.5 mmol^/^L AsA (NA); (4) 100 mmol/L NaCl and 0.25 mmol/L LYC (NL); and (5) 100 mmol^/^L NaCl, 0.25 mmol/L LYC, and 0.5 mmol/L AsA (NLA). AsA and LYC were purchased from Sigma (United States) and YuanYe (China), respectively.

The methods, volumes, and concentrations of LYC and AsA application were based on a prior experiment (Supplementary Materials). The containers were arranged in a randomized complete block with three replicates per treatment. Each container contains five plants. The seedlings were exposed to light for 14 h at temperatures of 24–30°C during daytime and 17–20°C during nighttime. The nutrient mixtures were replaced every third day. The samples of tomato seedling leaves were obtained on the 9th day of treatment.

### Growth Measurements

The following growth indices, such as plant height, stem diameter, shoot dry weight, root dry weight, root fresh weight, and shoot fresh weight, were measured. In details, the length from the cotyledonary internode to the growth point was measured and considered as the plant height; the diameter of cotyledonary node was measured and considered as the stem diameter of the seedling. Then, the seedlings were washed with tap water and wiped with paper. The shoot and root of each seedling were separated and weighed, dried for 15 min in a 105°C oven, and then to a constant weight at 75°C. Then, the samples were weighed.

### Determination of Photosynthetic Pigment

Fresh processing tomato leaves (0.1 g) were harvested from the different treatments and put into a 25 mL brown volumetric flask, mixed with a 10 mL acetone–ethanol mix (1:1), and placed in the dark at room temperature until turning white. The extracting reagent was used as a negative control. We measured the chlorophyll extract absorbance at 470, 646, and 663 nm and analyzed the concentration of photosynthetic pigments according to the following (Wellburn and Lichtenthaler, 1984): mass concentration of chlorophyll *a* (Chl*a*) (mg/g FW) = (12.21A_663_−2.81A_646_) × 10/(1,000 × 0.1); mass concentration of chlorophyll *b* (Chl*b*) (mg/g FW) = (20.13A_646_−5.03A_663_) × 10/(1,000 × 0.1); total mass concentration of chlorophyll (Chl*a* + *b*) (mg/g FW) = Chl*a* + Chl*b*; and mass concentration of carotenoids (Car) (mg/g FW) = (1,000A_470_−3.27 × Chl*a*-104 × Chl*b*) × 10/(1,000 × 0.1).

### Determination of Photosynthetic Parameters

Photosynthetic parameters measurements were performed using a portable photosynthetic system (Li6400, LI-COR, United States) at 9:00–11:00 h on the fully developed leaf of each plant, according to a method previously described by Diao et al. (2014). To measure the net photosynthetic rate (*P*_n_), intracellular CO_2_ concentration (*C*_i_), transpiration rate (*T*_r_), and stomatal conductance (*G*_s_), the air temperature, relative humidity, CO_2_ concentration, and PPFD were maintained at 28–32°C, 80%, 380–390 μmol/mol, and 1,500 μmol/m^2^/s, respectively, by automatic control device of the Li6400 photosynthetic system.

### Ascorbic Acid Contents

The total AsA and AsA content were determined following the procedure by Jiang and Zhang (2001) with slight modifications. Leaves (0.3 g) were homogenized in ice-cold 5% (w/v) trichloroacetic acid and centrifuged at 15,000 × *g* for 10 min. Half of each sample was assayed for total AsA, and the other half was assayed for AsA content only. The dehydroascorbate (DHA) content was calculated as the difference between the total AsA content and the AsA content.

### Measurement of OJIP Transients and Analysis of Fast Fluorescence Induction Kinetics

The chlorophyll (Chl) fluorescence transient (OJIP) curves were measured on fully expanded leaves that were still attached. Prior to these measurements, a leaf clip to subject five leaves to darkness for 30 min each was used. Then, the kinetics of Chl fluorescence (prompt fluorescence, PF) was measured, and the reflection at 820 nm (*I*/*I*_o_) with a plant efficiency analyzer (Handy PEA, Hansatech Instrument Ltd, Lynn, United Kingdom) at room temperature was modulated. When all of the PSII reaction centers (RCs) were open (O step), the minimum fluorescence intensity signal (*F*_o_) was assessed at 20 μs, and when all of the PSII RCs were close (P step), the maximum intensity signal (*F*_m_) was assessed at 200–500 ms (Strasser et al., 2004). The fluorescence intensities between these two outliers: 30 ms (I step), 3 ms (J step), 300 μs (K step), and 150 μs (L step) were designated as *F*_I_, *F*_J_, *F*_K_, and *F*_L_, respectively (Kalaji et al., 2017).

In order to perform a thorough assessment of the O–K and O–J periods, we normalized an original transient curve as a variable relative fluorescence according to the following: *W*_O−*K*_ = (*F*_t_−*F*_o_)/(*F*_K_−*F*_o_) and *W*_O−*J*_ = (*F*_t_−*F*_o_)/(*F*_J_−*F*_o_), respectively (Oukarroum et al., 2010; Ceppi et al., 2012). We analyzed the kinetic differences based on the variable relative fluorescence according to the following: Δ*W*_O−*K*_ = [*W*_O−*K*_(stress)–*W*_O−*K*_(control)] and Δ*W*_O−*J*_ = [*W*_O−*J*_(stress)–*W*_O−*J*_(control)], which determined the presence of the L- and K-bands, respectively (Dalberto et al., 2017).

The OJIP transient parameters were calculated according to the JIP test (Strasser et al., 2010). The formulas and their description of JIP test parameters are listed in Table 1.

**Table 1:** Summary of parameters and formulae using data extracted from chlorophyll (Chl) *a* fluorescence (OJIP) transient.

**Parameters**	**Explanation**
**Parameters using data extracted from OJIP transient**	
*F*_v_/*F*_m_ = (*F*_t_−*F*_o_)/(*F*_m_−*F*_o_)	The maximal photochemical efficiency of PSII
V_J_ = (*F*_J_−*F*_o_)/(*F*_m_−*F*_o_)	Relative variable fluorescence intensity at the J-step
V_I_ = (*F*_I_−*F*_o_)/(*F*_m_−*F*_m_)	Relative variable fluorescence intensity at the I-step
V_K_ = (*F*_K_−*F*_o_)/(*F*_m_−*F*_o_)	Relative variable fluorescence intensity at the K-step
W_k_ = (*F*_K_−*F*_o_)/(*F*_J_−*F*_o_)	Ratio of variable fluorescence F_K_ to the amplitude *F*_J_−*F*_o_
W_L_ = (*F*_L_−*F*_o_)/(*F*_K_−*F*_o_)	Ratio of variable fluorescence F_L_ to the amplitude *F*_K_−*F*_o_
S_m_ = (Area)/(*F*_m_−*F*_o_)	Normalized total complementary area
M_o_ = 4(*F*_300μ*s*_−*F*_o_)/(*F*_m_−**F**_o_)	Approximated initial slope of the fluorescence transient
**Flux ratios of PSII**	
*Φ*p_o_ = TR_o_/ABS = [1−(*F*_o_/*F*_m_)]	Maximum quantum yield of primary photochemistry
ϕE_o_= ET_o_/ABS = [1−(*F*_o_/*F*_m_)]ψ_o_	Quantum yield for electron transport (at *t* = *F*_o_)
ϕD_o_ = 1−ϕ_Po_ = (*F*_o_/*F*_m_)	Quantum yield at *t* = *F*_O_ for energy dissipation
ψ_o_ = ET_o_/TR_o_ = (1−V_J_)	Probability that a trapped exciton moves an electron into the electron transport chain beyond ${\text{Q}}_{{\text{A}}^{-}}$(at *t* = *F*_o_)
**Activities per reaction center (RC)**	
ABS/RC = M_o_(1/V_J_)(1/ϕ_Po_)	Absorption flux per RC
TR_o_/RC = M_o_(1/V_J_)	Trapped energy flux per RC (at *t* = *F*_o_)
DI_o_/RC = ABS/RC- TR_o_/RC	Dissipated energy flux per RC (at *t* = *F*_o_)
ET_o_/RC = M_o_(1/V_J_) (1/V_J_)	Electron transport flux per RC (at *t* = *F*_o_)
**Phenomenological energy fluxes per excited cross section (CS**_**m**_ **, subscript** ***M*** **refer to time** ***t*** **=** ***F***_**m**_ **):**	
ABS/CS_m_≈*F*_m_	Absorption flux per CS (at *t* = *F*m)
TR_o_/CS_m_ = *Φ*_po_ (ABS/CS_m_)	Trapped energy flux per CS (at *t* = *F*_m_)
DI_o_/CS_m_ = (ABS/CS_m_) - (TR_o_/CS_m_)	Dissipated energy flux per CS (at *t* = *F*_m_)
ET_o_/CS_m_ = *Φ*_Eo_ (ABS/CS_m_)	Electron transport flux per CS (at t = *F*_m_)
**Flux ratios of PS I**	
δR_o_= RE_o_/ET_o_ = (1−V_I_)/(1–V_J_)	Efficiency with which an electron from the intersystem electron carriers is transferred to reduce end electron acceptors at the PSI acceptor side
ϕR_o_ = RE/ABS = TR_o_/ABS (1–V_I_)	Quantum yield for reduction of end electron acceptors at the PSI acceptor side
ΔI/I_o_ = (I_max_−I_min_)/I_o_	Maximal redox capacity of PSI
**Vitality indexes**	
*PI*_ABS_ = RC/ABS [ϕP_o_/(1–ϕP_o_)] [ψ_o_/(1–ψ_o_)]	Performance index on absorption basis

### Statistical Analysis

All data are presented as means ± standard deviation (SD) of three replicates. Statistical analyses were performed by the analysis of variance (ANOVA) using SPSS version 19.0 (SPSS Inc., Chicago, IL, United States). The significant differences among treatments were analyzed by the Duncan's multiple range test at a 0.05 probability level. Differences at *P* < 0.05 were considered to be significant.

## Results

### Plant Growth

In comparison with the control treatment, plant height, stem diameter, dry and fresh weights of root and shoot were decreased by 31.9%, 25.5%, 66.1%, 45.8%, 74.2%, and 73.8% (*P* < 0.05) in the leaves of tomato plants under salt stress, respectively.

**Figure 1:**
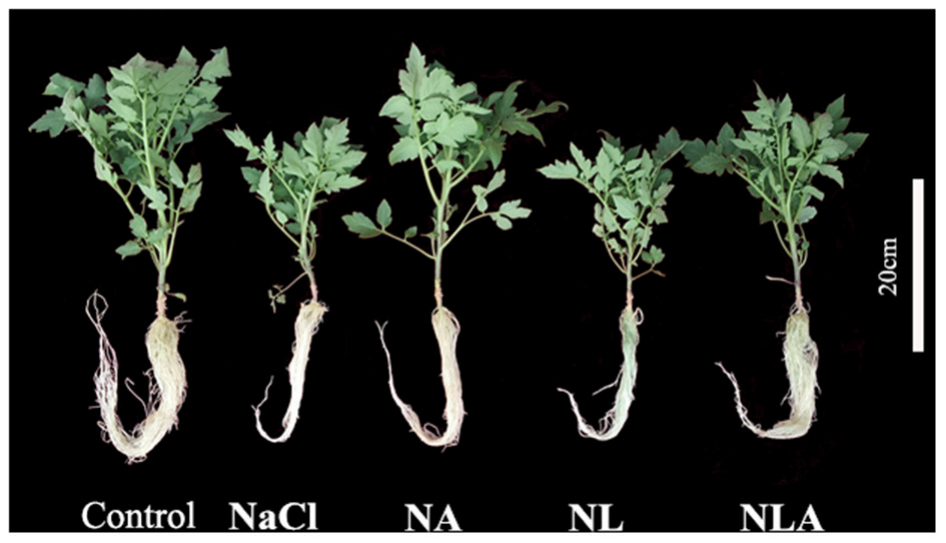
Growth of salt-stressed tomato seedlings with or without exogenous ascorbic acid (AsA) and lycorine (LYC) spraying. Control, no added NaCl, no sprayed AsA and LYC; NaCl, added 100 mmol/L NaCl; NA, added 100 mmol/L NaCl and sprayed 0.5 mmol/L AsA; NL, added 100 mmol/L NaCl and sprayed 0.25 mmol/L LYC; NLA, added 100 mmol/L NaCl and sprayed 0.25 mmol/L LYC and 0.5 mmol/L AsA.

Lycorine (LYC) spraying (NL treatment) significantly reduces those effects on plant height, stem diameter, and fresh weights of shoot, but not on dry weight of shoot and fresh weight of root. However, exogenous application of AsA can mitigate NaCl and LYC effects (NA and NLA treatments) (Table 2; Figure 1).

**Table 2:** The plant height (cm), stem diameter (mm), shoot FW (g), and root FW (g), shoot DW (g), and root DW (g) in the leaves of salt-stressed tomato seedlings as affected by exogenous ascorbic acid (AsA) and lycorine (LYC).

**Treatment**	**Plant height**	**Stem diameter**	**Shoot FW**	**Root FW**	**Shoot DW**	**Root DW**
Control	21.43 ± 0.76a	8.58 ± 0.53a	57.65 ± 1.04a	24.97 ± 5.92a	5.24 ± 0.32a	1.71 ± 0.09a
NaCl	14.60 ± 0.26d	6.39 ± 0.16c	15.10 ± 0.49c	13.55 ± 0.28c	1.35 ± 0.15c	0.58 ± 0.07d
NA	16.97 ± 0.47b	7.69 ± 0.41b	41.84 ± 0.71b	19.14 ± 1.50b	2.14 ± 0.28b	0.94 ± 0.07b
NL	12.37 ± 0.42e	5.60 ± 0.19d	13.11 ± 0.48d	10.00 ± 0.36c	1.27 ± 0.07c	0.55 ± 0.03d
NLA	15.70 ± 0.72c	6.44 ± 0.40c	14.81 ± 0.43c	14.23 ± 0.64bc	1.59 ± 0.06bc	0.76 ± 0.04c

*Different letters (a, b, c, d, and e) indicate significant difference at P < 0.05 among treatments. Note: Control, no added NaCl, no sprayed AsA and LYC; NaCl, added 100 mmol/L NaCl; NA, added 100 mmol/L NaCl and sprayed 0.5 mmol/L AsA; NL, added 100 mmol/L NaCl and sprayed 0.25 mmol/L LYC; NLA, added 100 mmol/L NaCl and sprayed 0.25 mmol/L LYC and 0.5 mmol/L AsA*.

### Endogenous Ascorbic Acid Content and Redox State

**Figure 2:**
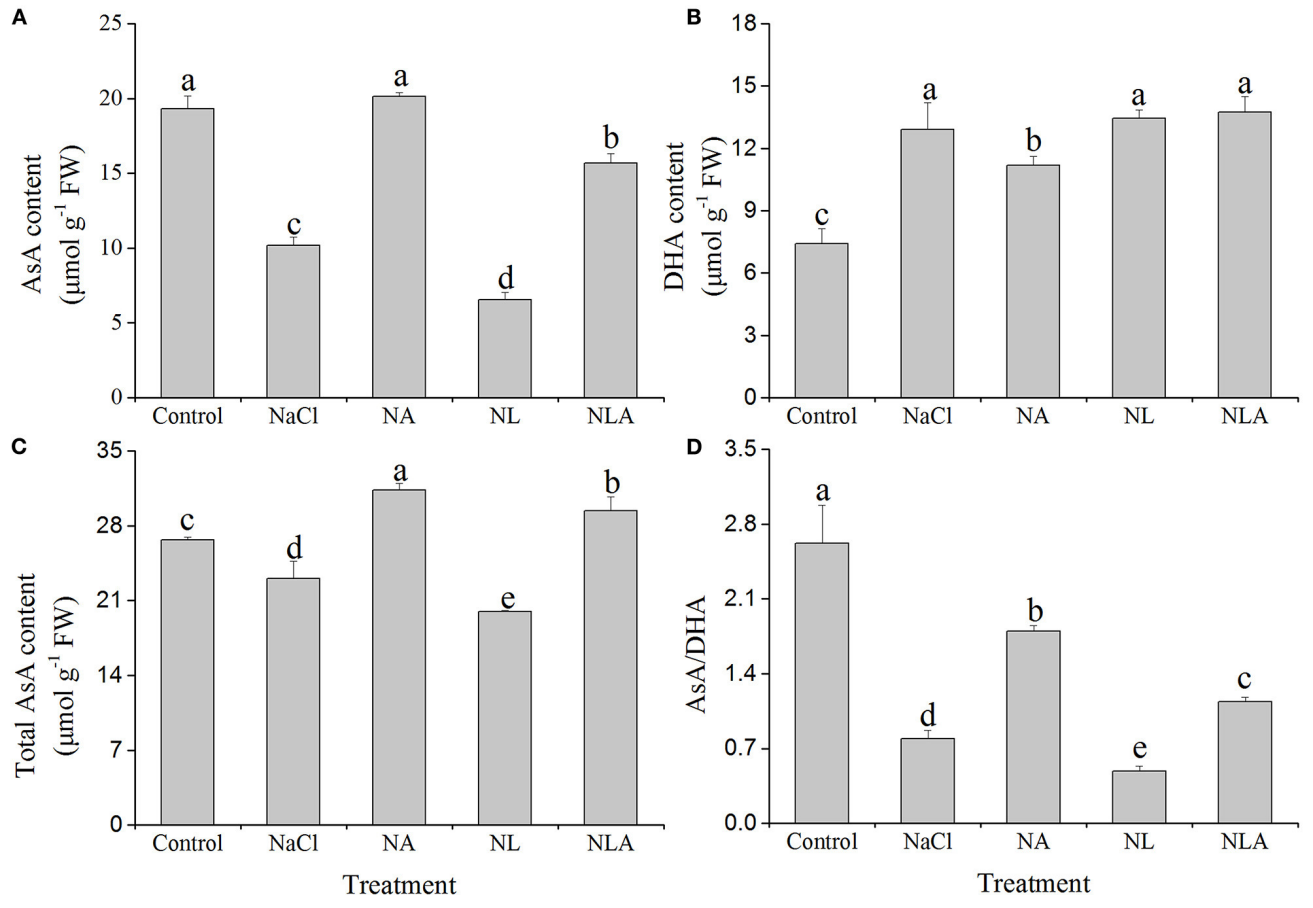
Values of ascorbic acid (AsA) content **(A)**, dehydroascorbate (DHA) content **(B)**, total AsA content **(C)**, and AsA/DHA **(D)** in the leaves of salt-stressed tomato seedlings with or without exogenous AsA and lycorine (LYC) spraying. Values are means ± SD (*n* = 3). Different letters (a, b, c, d, and e) indicate significant difference at *P* < 0.05 among treatments. Control, no added NaCl, no sprayed AsA and LYC; NaCl, added 100 mmol/L NaCl; NA, added 100 mmol/L NaCl and sprayed 0.5 mmol/L AsA; NL, added 100 mmol/L NaCl and sprayed 0.25 mmol/L LYC; NLA, added 100 mmol/L NaCl and sprayed 0.25 mmol/L LYC and 0.5 mmol/L AsA.

Both the total AsA and AsA contents and the AsA/dehydroascorbate (DHA) ratio are considerably lower compared with the control under NaCl stress, which the AsA spraying increases. On the contrary, the DHA content is increased compared with the control (Figure 2). Compared with NaCl treatment, the AsA application to NaCl-stressed plants increases the content of total AsA, AsA, and AsA/DHA ratio by 26.3%, 49.4%, and 56.1% (*P* < 0.05), respectively, and the DHA content is decreased by 13.3% (*P* < 0.05). The AsA application to NL-treated plants also significantly increases the contents of total AsA and AsA and the ratio of AsA/DHA, but has no influence on the DHA level (Figure 2).

### Photosynthetic Pigments Content

Tomato plants submitted to salt stress show a considerable decline in the chlorophyll (Chl) content, carotenoids (Car) content, and Car/Chl*a*+*b* ratio, whereas the Chl*a*/*b* ratio increased compared with the control. The AsA application to NaCl-stressed plants increases the content of Chl*a*, Chl*b*, Chl*a*+*b*, and Car by 28.0%, 52.4%, 33.3%, and 69.41% (*P* < 0.05), respectively, compared with the NaCl treatment. The LYC application to salt-stressed plants (NL-treatment) accentuates the negative effects of NaCl stress on photosynthetic pigments content, but AsA application (NLA-treatment) can attenuate them (Table 3).

**Table 3:** The chlorophyll content (mg/g FW), Chl*a*/*b* ratio, carotenoids content (mg/g FW), and Car/Chl*a*+*b* ratio in the leaves of salt-stressed tomato seedlings as affected by exogenous acid (AsA) and lycorine (LYC).

**Treatment**	**Chlorophyll (Chl) content**			**Chl*a/b***	**Carotenoids (Car) content**	**Car/Chl*a+b***
	**Chl*a***	**Chl*b***	**Chl*a* + Chl*b***			
Control	0.89 ± 0.02b	0.36 ± 0.01a	1.25 ± 0.02a	2.48 ± 0.13d	74.36 ± 0.26b	59.39 ± 1.33c
NaCl	0.75 ± 0.01d	0.21 ± 0.02c	0.96 ± 0.01c	3.60 ± 0.13ab	47.56 ± 1.35c	49.47 ± 1.74e
NA	0.96 ± 0.02a	0.32 ± 0.01b	1.28 ± 0.03a	3.04 ± 0.07c	80.57 ± 1.72a	63.01 ± 2.50b
NL	0.57 ± 0.04e	0.16 ± 0.01d	0.74 ± 0.05d	3.47 ± 0.03b	40.44 ± 3.08d	54.38 ± 0.32d
NLA	0.81 ± 0.01c	0.21 ± 0.05c	1.03 ± 0.07b	3.75 ± 0.15a	74.82 ± 2.05b	72.32 ± 2.21a

*Values are means ± SD (n = 3). Different letters (a, b, c, d, and e) indicate significant difference at P < 0.05 among treatments. Note: Control, no added NaCl, no sprayed AsA and LYC; NaCl, added 100 mmol/L NaCl; NA, added 100 mmol/L NaCl and sprayed 0.5 mmol/L AsA; NL, added 100 mmol/L NaCl and sprayed 0.25 mmol/L LYC; NLA, added 100 mmol/L NaCl and sprayed 0.25 mmol/L LYC and 0.5 mmol/L AsA*.

### Gas Exchange

**Figure 3:**
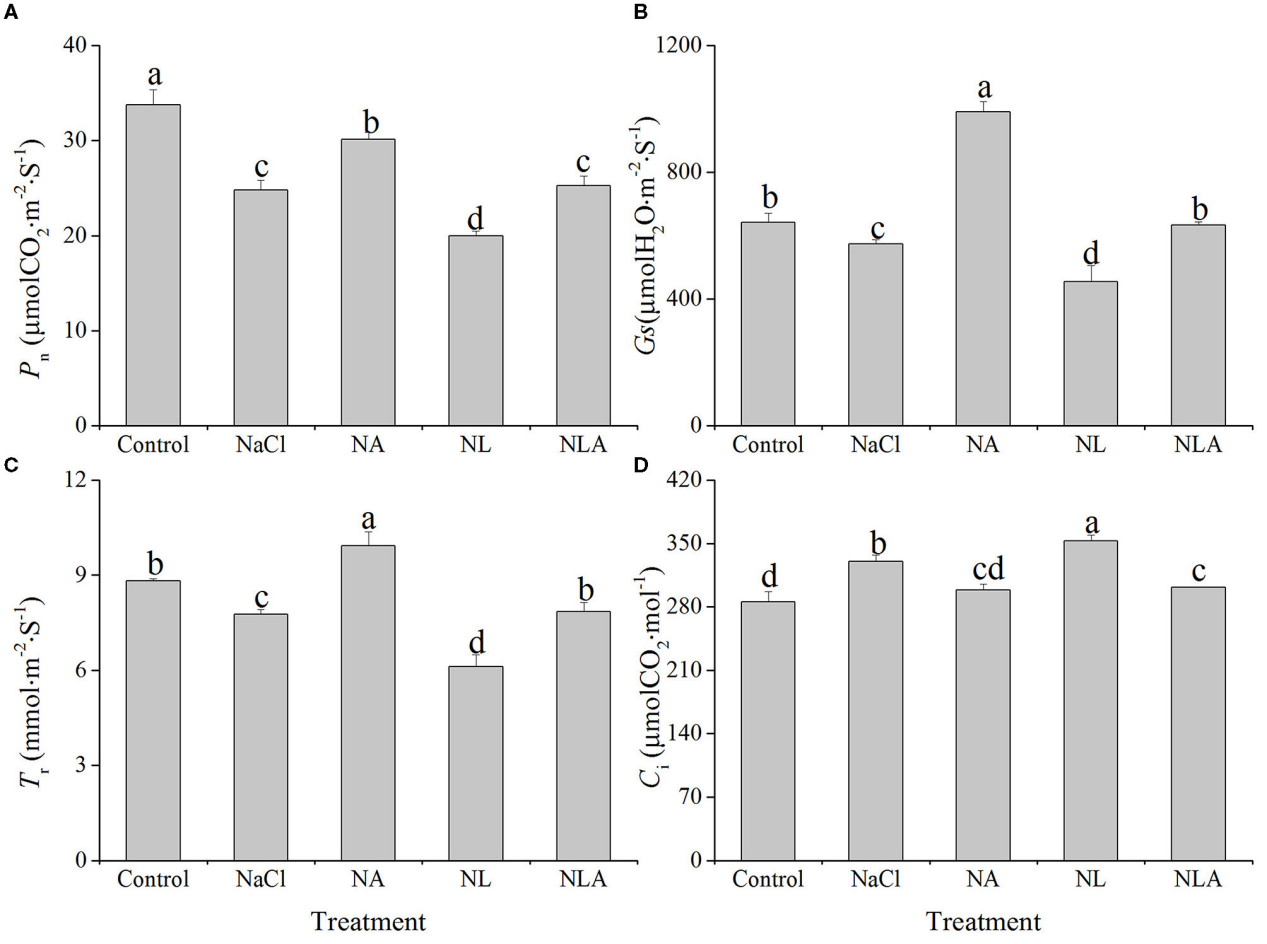
Values of net photosynthetic rate (P_n_) **(A)**, stomatal conductance (G_s_) **(B)**, transpiration rate (_Tr_) **(C)**, and intercellular CO_2_ concentration (C_i_) **(D)** in the leaves of salt-stressed tomato seedlings with or without exogenous ascorbic acid (AsA) and lycorine (LYC) spraying. Values are means ± SD (*n* = 3). Different letters (a, b, c, and d) indicate significant difference at *P* < 0.05 among treatments. Control, no added NaCl, no sprayed AsA and LYC; NaCl, added 100 mmol/L NaCl; NA, added 100 mmol/L NaCl and sprayed 0.5 mmol/L AsA; NL, added 100 mmol/L NaCl and sprayed 0.25 mmol/L LYC; NLA, added 100 mmol/L NaCl and sprayed 0.25 mmol/L LYC and 0.5 mmol/L AsA.

In comparison with the control treatment, the net photosynthetic rate (*P*_n_), stomatal conductance (*G*_s_), and transpiration rate (*T*_r_) are respectively and significantly decreased by 26.6%, 10.6%, and 11.9%, whereas the intercellular CO_2_ concentration (*C*_i_) is increased by 10.6% (*P* < 0.05) in the leaves of tomato plants under salt stress. The application of LYC further decreases *P*_n_, *G*_s_, and *T*_r_ under salt stress. However, the application of AsA significantly alleviates the reduction of leaf *P*_n_, *G*_s_, and *T*_r_ induced by NaCl and NL treatments. *C*_i_ values in tomatoes under NaCl treatment and NL treatment are decreased after AsA spraying (NA and NLA conditions) (Figure 3).

### OJIP Transient Curves

**Figure 4:**
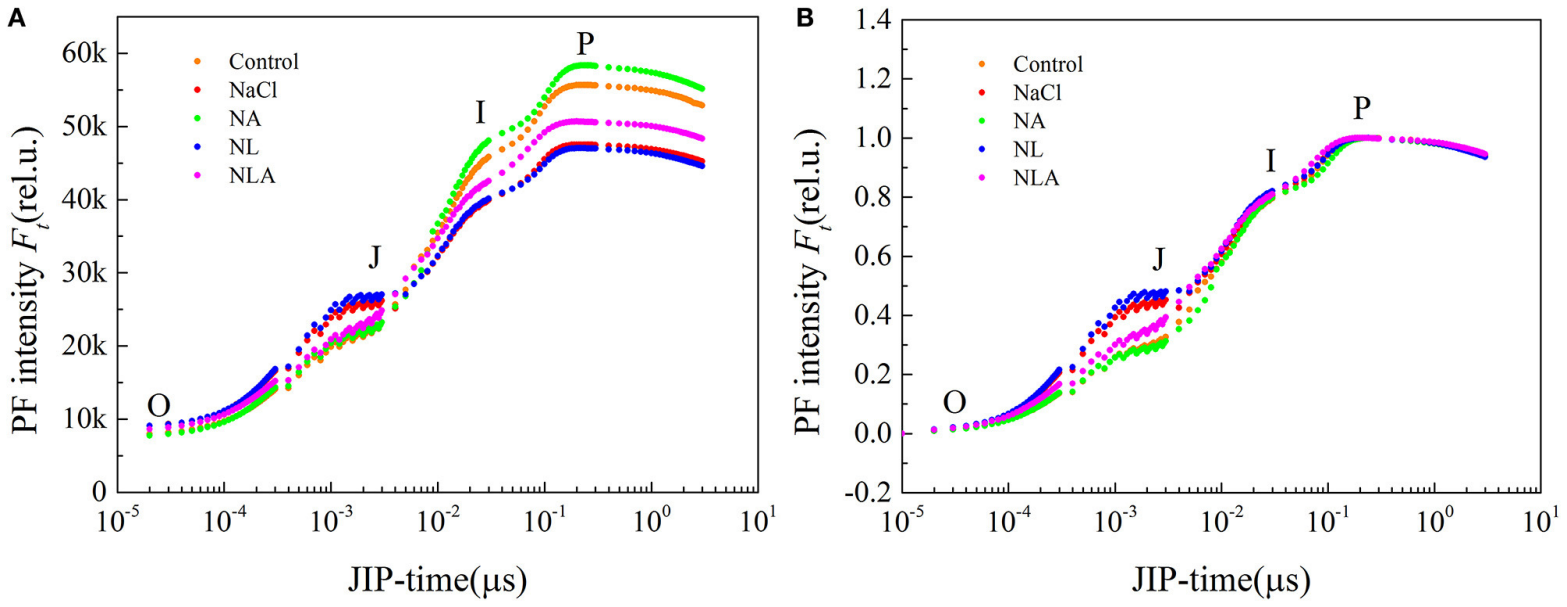
Prompt fluorescence (PF) curves **(A)** and its normalized curves between O and P steps expressed as *W*_O−*P*_ = [(*F*_t_−*F*_o_)/(*F*_m_−*F*_o_)] **(B)** in the leaves of salt-stressed tomato seedlings with or without exogenous ascorbic acid (AsA) and lycorine (LYC) spraying. Values are means ± SD (*n* = 3). Control, no added NaCl, no sprayed AsA and LYC; NaCl, added 100 mmol/L NaCl; NA, added 100 mmol/L NaCl and sprayed 0.5 mmol/L AsA; NL, added 100 mmol/L NaCl and sprayed 0.25 mmol/L LYC; NLA, added 100 mmol/L NaCl and sprayed 0.25 mmol/L LYC and 0.5 mmol/L AsA.

The OJIP transient curves between O and P phases are presented in Figure 4A. As illustrated in the figure, the NaCl treatment markedly increases J phase, but decreases I and P phases, and the amplitude of I–P phase. The NA treatment reverses salt-induced changes in OJIP curve, with an even higher PF intensity during I and P phases and a greater amplitude of I–P phase than that in the control. In the NL-treated leaves, the PF intensity during O and J phases is higher than that in the NaCl-treated leaves, but the I and P phases are not affected. The NLA-treated plants show lower PF intensity during the J phase and a higher one during the I and P phases compared with the NL-treated seedlings.

We normalized the fluorescence data and displayed it as the kinetics of relative variable fluorescence at any time *W*_O−*P*_ = (*F*_t_−*F*_o_)/(*F*_m_−*F*_o_) (Figure 4B) to assess the polyphasic processes of the OJIP curves for the processes observed in the O–J, J–I, and I–P phases. Assessing *W*_O−*P*_ helped identify the sites where various electron transport chains on the acceptor end of PSII were treated (Kalaji et al., 2017). Figure 4B shows that there are no variations of *W*_O−*P*_ in both J–I and I–P phases between all treatments. However, in the O–J phase, a strong increase of PF intensity under salt stress is observed in comparison with the control, enhanced by LYC application. However, the AsA spraying has the opposite effect of LYC on both NaCl- and NL-treated leaves with a decrease in PF intensity during the O–J phase (Figure 4B).

**Figure 5:**
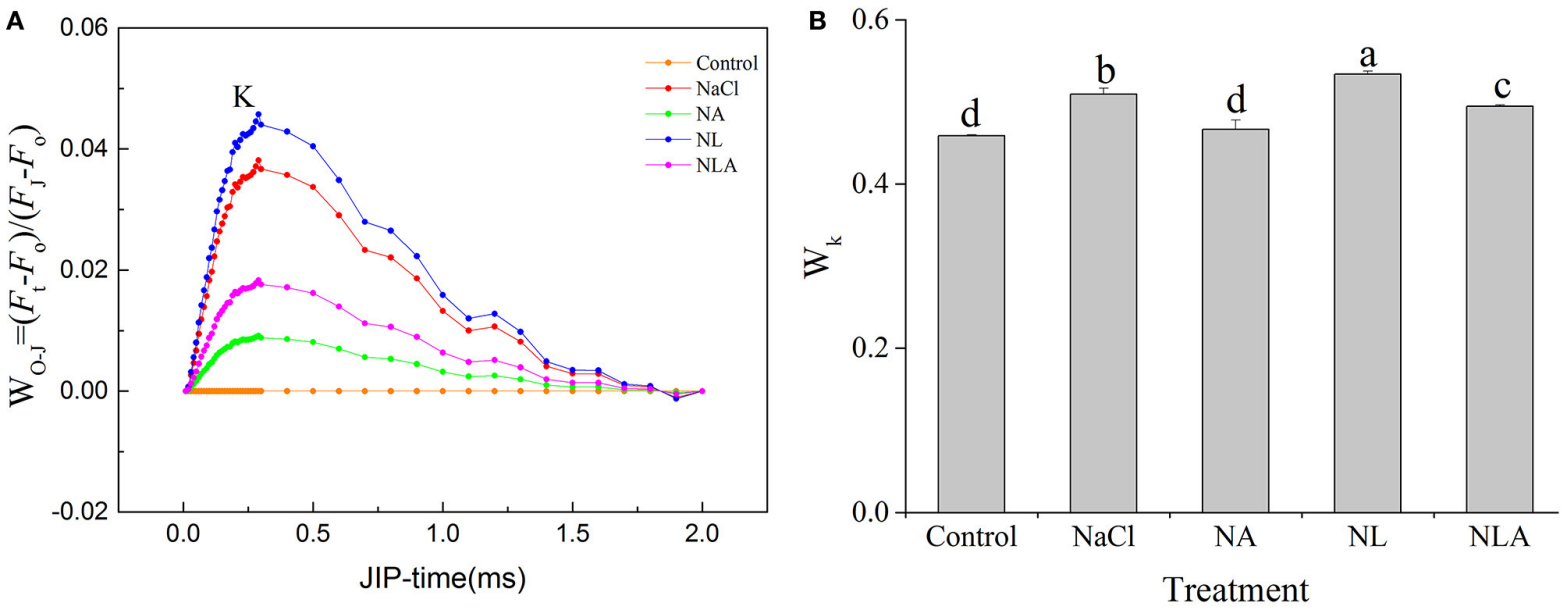
Normalized prompt fluorescence (PF) curves between O and J steps expressed as *W*_O−*J*_ = [(*F*_t_−*F*_o_)/(*F*_J_−*F*_o_)] **(A)** and ratio of variable fluorescence *F*_K_ to the amplitude *F*_J_−*F*_O_ expressed as *W*_K_ = (*F*_K_−*F*_o_)/(*F*_J_−*F*_o_) **(B)** in the leaves of salt-stressed tomato seedlings with or without exogenous ascorbic acid (AsA) and lycorine (LYC) spraying. Values are means ± SD (*n* = 3). Different letters (a, b, c, and d) indicate significant difference at *P* < 0.05 among treatments. Control, no added NaCl, no sprayed AsA and LYC; NaCl, added 100 mmol/L NaCl; NA, added 100 mmol/L NaCl and sprayed 0.5 mmol/L AsA; NL, added 100 mmol/L NaCl and sprayed 0.25 mmol/L LYC; NLA, added 100 mmol/L NaCl and sprayed 0.25 mmol/L LYC and 0.5 mmol/L AsA.

The K-band appearing at 300 s reflects, when positive, a degradation or inactivation of the oxygenation complex (OEC) and is the specific marker of photoinhibition on the PSII receptor side (Yusuf et al., 2010). In order to observe K-band, the relative fluorescence between the phases O and J was normalized (Figure 5A), and the ratios of variable fluorescence *F*_K_ to the amplitude *F*_J_−*F*_o_ (*W*_K_) were calculated (Figure 5B). Under salt stress, K-band and *W*_K_ are significantly higher than that in the control, with further increase by the application of LYC. Exogenous foliar spray of AsA in the NaCl treatment and the NL treatment alleviates this increase.

**Figure 6:**
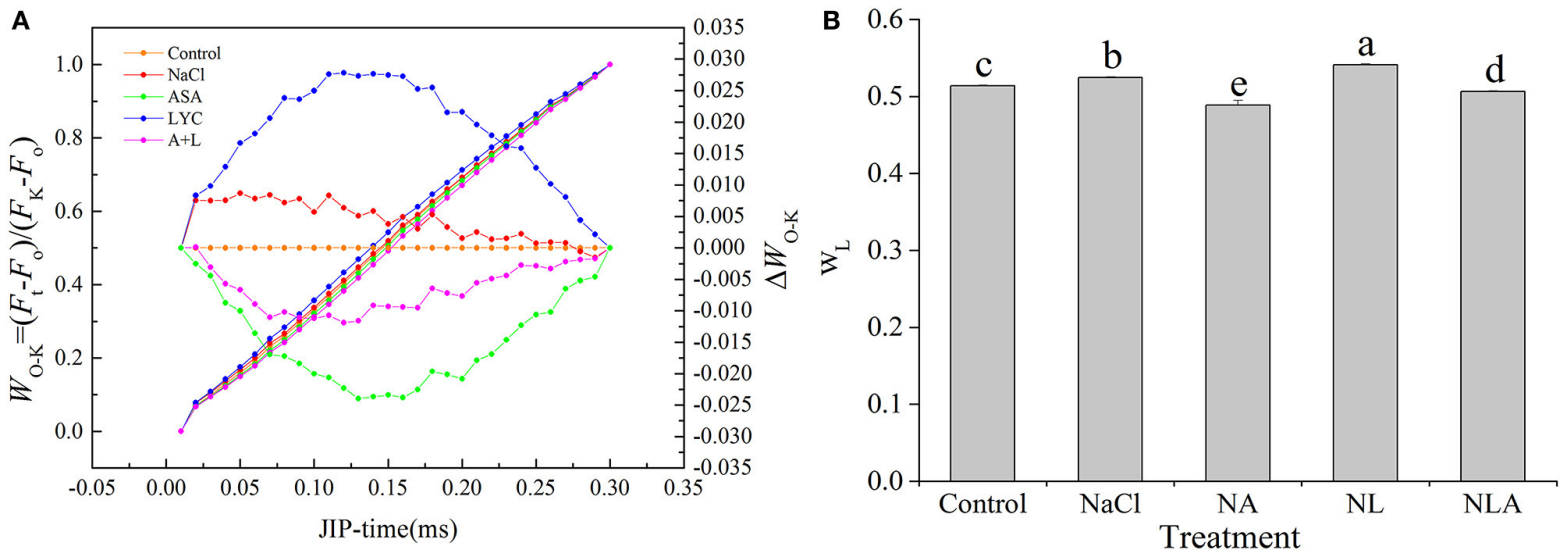
Shape of the prompt fluorescence (PF) transient curves normalized between O and K steps expressed as *W*_O−*K*_ = [(*F*_t_−*F*_o_)/(*F*_K_−*F*_o_)], Δ*W*_O−*K*_ = *W*_O−*K*_(stress)–*W*_O−*K*_ (control) **(A)**, *W*_L_ = [(*F*_L_−*F*_o_)/(*F*_K_−*F*_o_)] **(B)** in leaves of salt-stressed tomato seedling with or without exogenous reduced ascorbic acid (AsA) and lycorine (LYC) spraying. Values are means ± SD (*n* = 3). Different letters (a, b, c, d, and e) indicate significant difference at *P* < 0.05 among treatments. Error bar is smaller than the line at the top of the graph. Control, no added NaCl, no sprayed AsA and LYC; NaCl, added 100 mmol/L NaCl; NA, added 100 mmol/L NaCl and sprayed 0.5 mmol/L AsA; NL, added 100 mmol/L NaCl and sprayed 0.25 mmol/L LYC; NLA, added 100 mmol/L NaCl and sprayed 0.25 mmol/L LYC and 0.5 mmol/L AsA.

The L-band appears at 150 μs, and its rise is used as a specific marker for thylakoid dissociation. We normalized the relative fluorescence between the O and K phases (*W*_O−*K*_) (Figure 6A, left), displaying them as the kinetic difference Δ*W*_O−*K*_ (Figure 6A, right). The kinetic difference Δ*W*_O−*K*_ makes the L-band visible. As shown in Figures 6A,B, exposure to salt stress leads to higher L-band and *W*_L_ in the leaves compared with the control plants. This increase is significantly higher after the LYC application. AsA-treated plants (both NA and NLA treatments) have significantly lower L-band and *W*_L_ (*P* < 0.05).

### JIP Test Parameters

#### PSII

**Figure 7:**
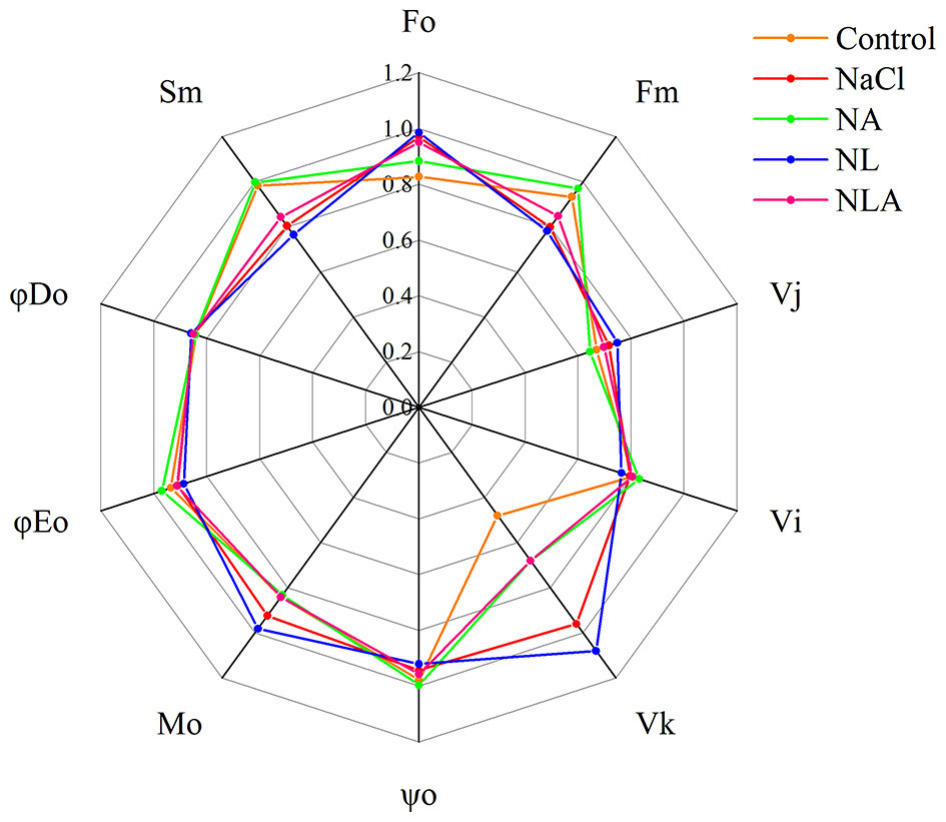
Spider plots of the JIP parameters deduced from chlorophyll a fluorescence OJIP transient curves in leaves of salt-stressed tomato seedlings with or without exogenous ascorbic acid (AsA) and lycorine (LYC) spraying. Values are means ± SD (*n* = 3). Control, no added NaCl, no sprayed AsA and LYC; NaCl, added 100 mmol/L NaCl; NA, added 100 mmol/L NaCl and sprayed 0.5 mmol/L AsA; NL, added 100 mmol/L NaCl and sprayed 0.25 mmol/L LYC; NLA, added 100 mmol/L NaCl and sprayed 0.25 mmol/L LYC and 0.5 mmol/L AsA.

The values of the JIP test parameters (presented in Table 1) are shown as a spider plot in Figure 7. In the leaves of seedlings under NaCl treatment, the values of ψ_o_, ϕ*E*_o_, S_m_, *F*_m_, and *V*_I_ are lower than that in the control, whereas the values of ϕ*D*_o_, *M*_o_, *F*_o_, *V*_K_, and *V*_J_ are higher than that in the control. In the JIP parameters of NL-treated plants, the values of ψ_o_, ϕ*E*_o_, *S*_m_, *F*_m_, and *V*_I_ are lower and the values of ϕ*D*_o_, *M*_o_, *F*_o_, *V*_K_, and *V*_J_ are higher than that under NaCl treatment. Conversely, the AsA-treated plants with NaCl treatment have higher ψ_o_, ϕ*E*_o_, *S*_m_, *F*_m_, and *V*_I_ and lower ϕ*D*_o_, *M*_o_, *F*_o_, *V*_K_, and *V*_J_ than that under NaCl treatment. The AsA application to NL-treated plants also increased the values of ψ_o_, ϕ*E*_o_, *S*_m_, *F*_m_, and *V*_I_ and decreased the values of ϕ*D*_o_, *M*_o_, *F*_o_, *V*_K_, and *V*_J_.

**Figure 8:**
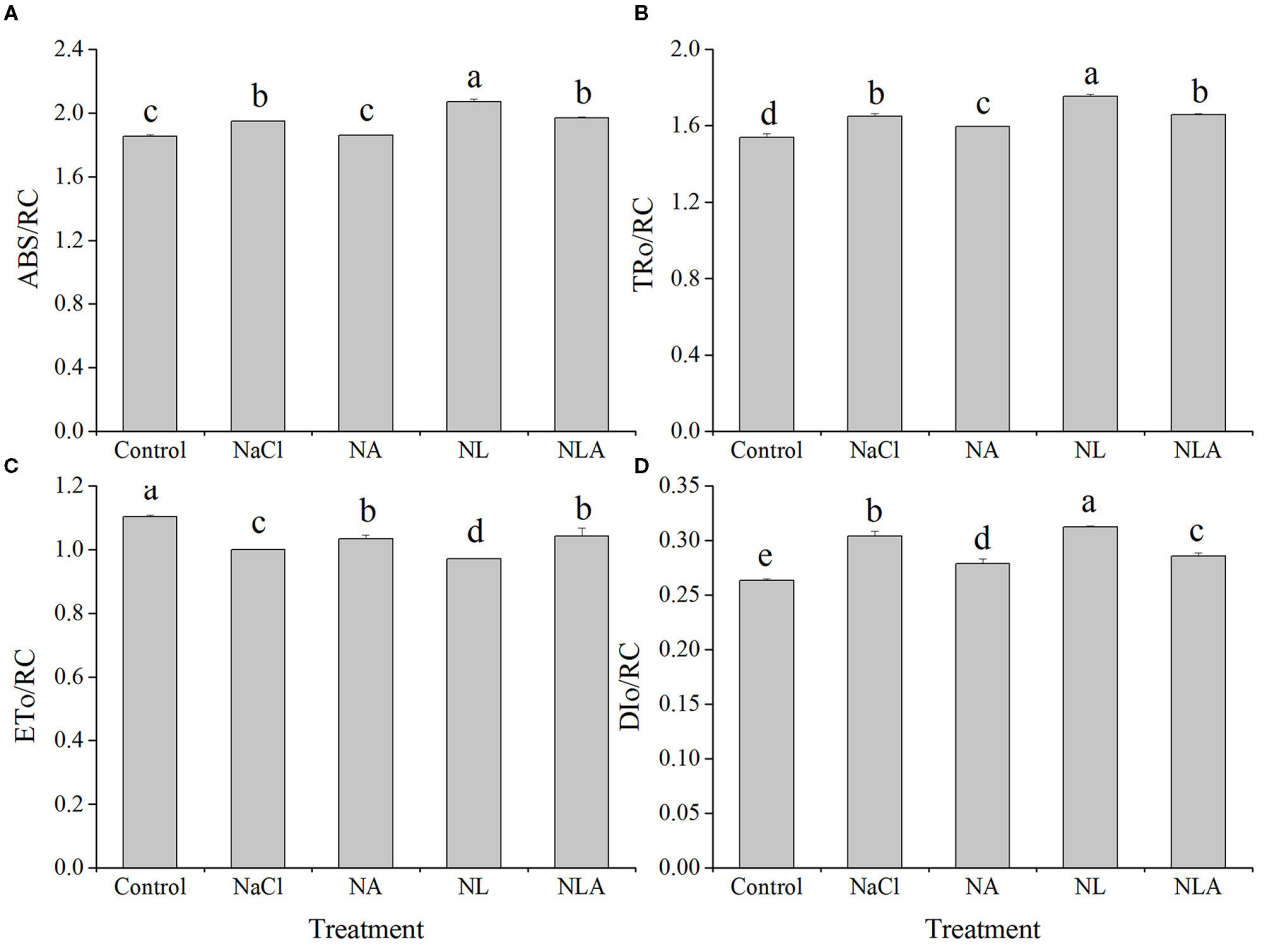
Parameters of absorption flux per reaction center (RC) (ABS/RC) **(A)**, trapped energy flux per RC (at *t* = *F*_o_) (TR_o_/RC) **(B)**, electron transport flux per RC (at *t* = *F*_o_) (ET_o_/RC) **(C)**, and dissipated energy flux per RC (at *t* = *F*_o_) (DI_o_/RC) **(D)** in leaves of salt-stressed tomato seedling with or without exogenous ascorbic acid (AsA) and lycorine (LYC) spraying. Values are means ± SD (*n* = 3). Different letters (a, b, c, d, and e) indicate significant difference at *P* < 0.05 among treatments. Error bar is smaller than the line at the top of the graph. Control, no added NaCl, no sprayed AsA and LYC; NaCl, added 100 mmol/L NaCl; NA, added 100 mmol/L NaCl and sprayed 0.5 mmol/L AsA; NL, added 100 mmol/L NaCl and sprayed 0.25 mmol/L LYC; NLA, added 100 mmol/L NaCl and sprayed 0.25 mmol/L LYC and 0.5 mmol/L AsA.

As shown in Figure 8, ABS/RC, TR_o_/RC, and DI_o_/RC (presented in Table 1) in the leaves of tomatoes under salt stress increased significantly compared with the control treatment, but ET_o_/RC decreased markedly. Compared with the NaCl treatment, ABS/RC, TR_o_/RC, and DI_o_/RC under NL treatment also increased significantly but ET_o_/RC decreased notably. However, the AsA spraying decreases ABS/RC, TR_o_/RC, and DI_o_/RC but increases ET_o_/RC with or without LYC.

**Figure 9:**
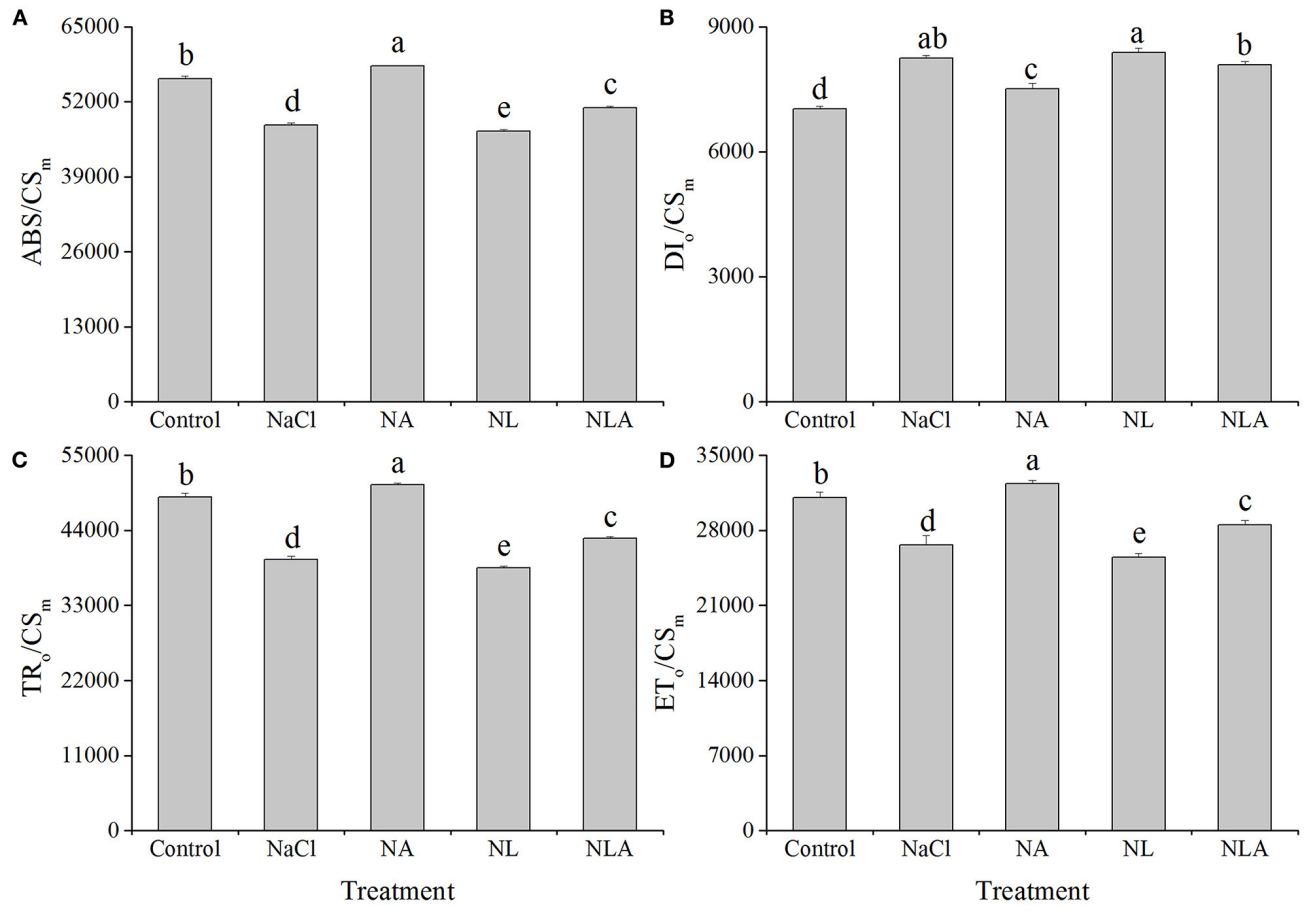
Parameters of absorption flux per CS (at *t* = *F*_m_) (ABS/CS_m_) **(A)**, dissipated energy flux per CS (at *t* = *F*_m_) (DI_o_/CS_m_) **(B)**, trapped energy flux per CS (at *t* = *F*_m_) (TR_o_/CS_m_) **(C)**, and electron transport flux per CS (at *t* = *F*_m_) (ET_o_/CS_m_) **(D)** in leaves of salt-stressed tomato seedlings with or without exogenous reduced ascorbic acid (AsA) and lycorine (LYC) spraying. Values are means ± SD (*n* = 3). Different letters (a, b, c, d, and e) indicate significant difference at *P* < 0.05 among treatments. Error bar is smaller than the line at the top of the graph. Control, no added NaCl, no sprayed AsA and LYC; NaCl, added 100 mmol/L NaCl; NA, added 100 mmol/L NaCl and sprayed 0.5 mmol/L AsA; NL, added 100 mmol/L NaCl and sprayed 0.25 mmol/L LYC; NLA, added 100 mmol/L NaCl and sprayed 0.25 mmol/L LYC and 0.5 mmol/L AsA.

As presented in Figure 9, ABS/CS_m_, TR_o_/CS_m_, and ET_o_/CS_m_ decreased by 14.3%, 18.9%, and 14.1%, respectively, in tomato leaves under salt stress, and DI_o_/CS_m_ increased significantly by 17.2% (*P* < 0.05), further decreasing with LYC application. However, the AsA application to NaCl- and NL-treated plants increases ABS/CS_m_, TR_o_/CS_m_ and ET_o_/CS_m_ significantly and decreases DI_o_/CS_m_ in both conditions.

**Figure 10:**
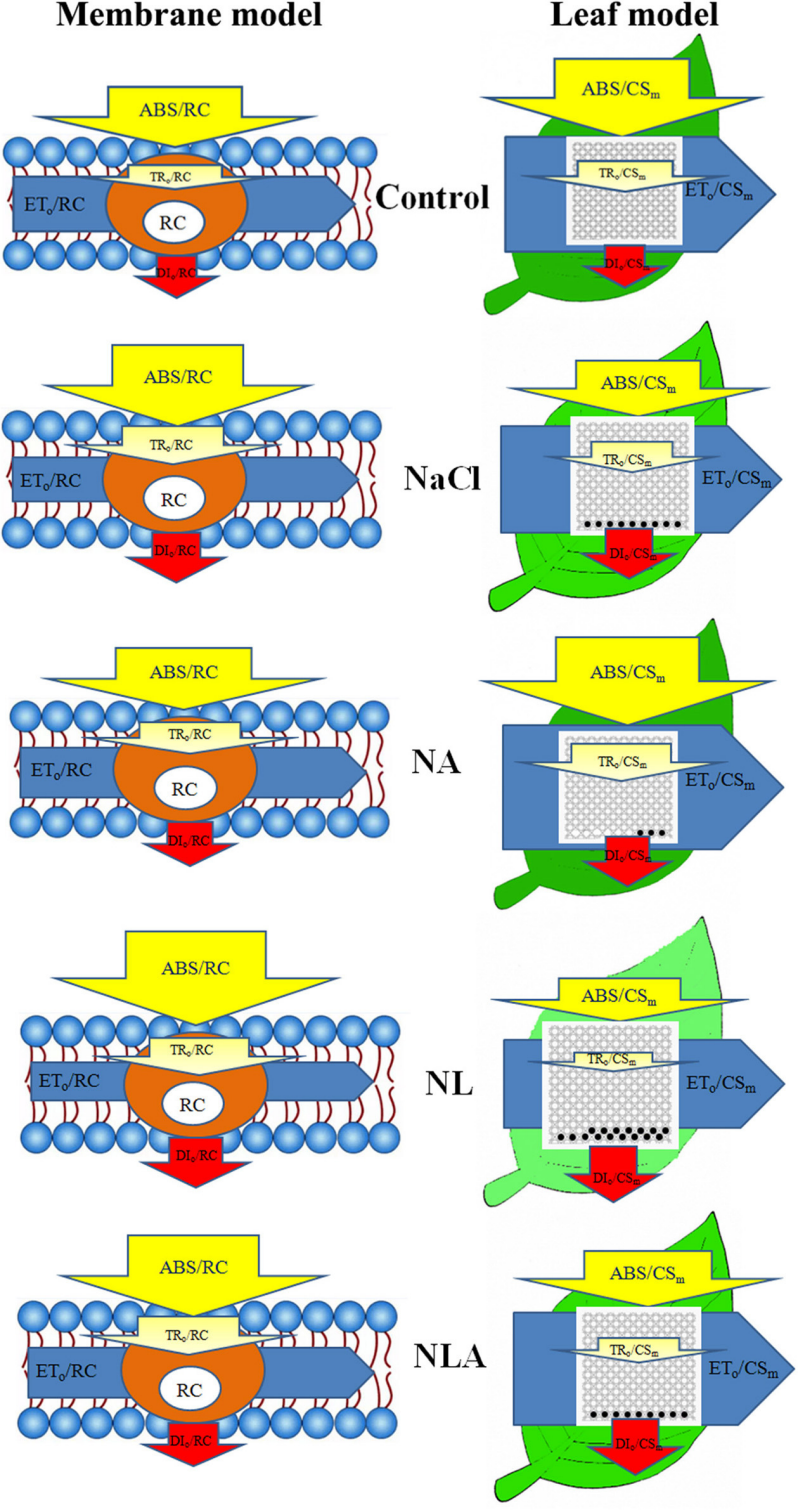
Energy pipeline models of specific fluxes per reaction center (RC) and phenomenological fluxes per excited cross-section (CS_m_) in the member (left) and the leaf (right) of salt-stressed tomato seedling with or without exogenous ascorbic acid (AsA) and lycorine (LYC) spraying. In the member and leaf models, ABS, TRo, ETo, and DIo indicate absorption, maximum trapping flux beyond Q_A_, electron transport, and dissipation flux, respectively. In the leaf models (right), the leaf color represents the pigment concentration, and the active and inactive RCs are indicated by open circles and closed circles, respectively. The detailed calculation of each parameter is given in Table 1. Control, no added NaCl, no sprayed AsA and LYC; NaCl, added 100 mmol/L NaCl; NA, added 100 mmol/L NaCl and sprayed 0.5 mmol/L AsA; NL, added 100 mmol/L NaCl and sprayed 0.25 mmol/L LYC; NLA, added 100 mmol/L NaCl and sprayed 0.25 mmol/L LYC and 0.5 mmol/L AsA.

**Figure 11:**
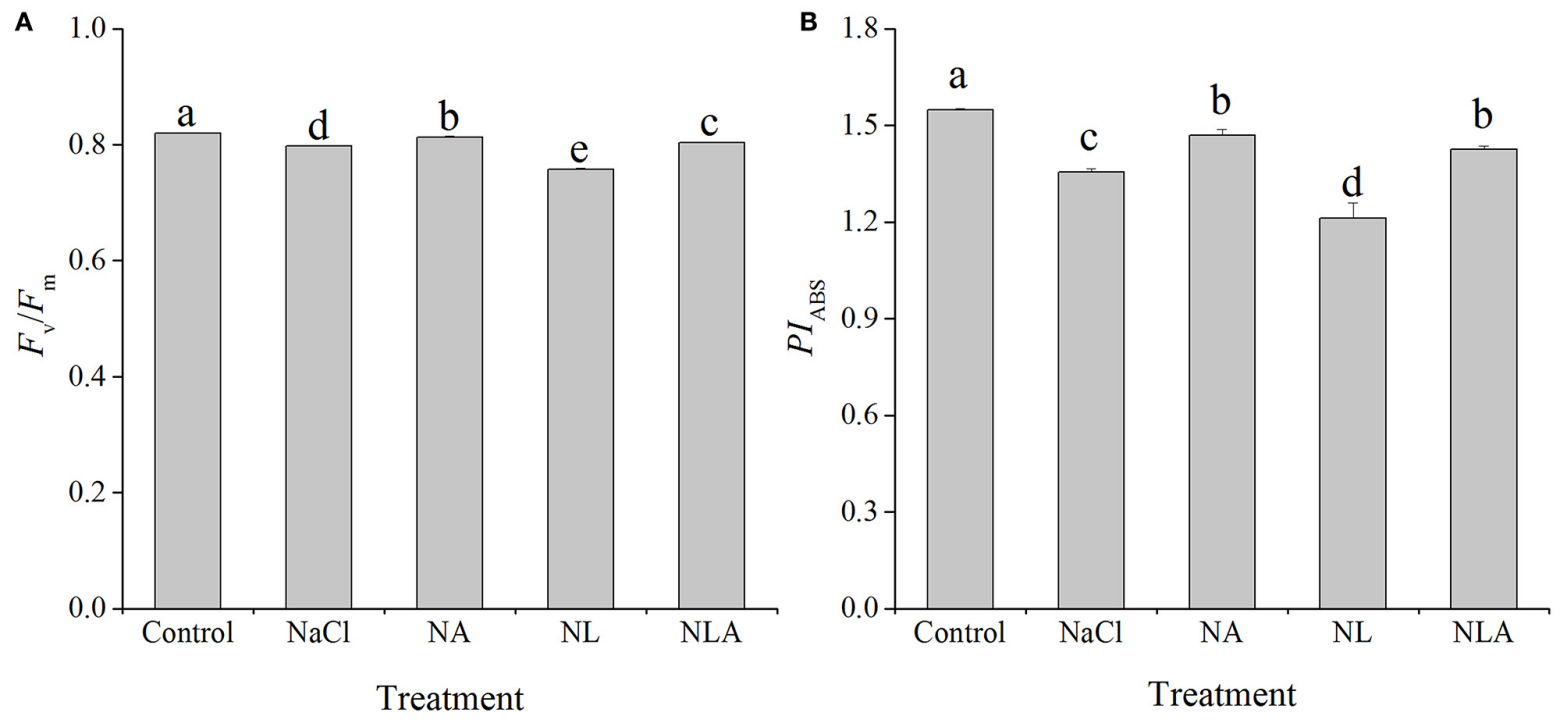
Values of the maximal photochemical efficiency of PSII (*F*_v_/*F*_m_) **(A)** and performance index on absorption basis (*PI*ABS) **(B)** in the leaves of salt-stressed tomato seedlings with or without exogenous ascorbic acid (AsA) and lycorine (LYC) spraying. Values are means ± SD (*n* = 3). Different letters (a, b, c, d, and e) indicate significant difference at *P* < 0.05 among treatments. Error bar is smaller than the line at the top of the graph. Control, no added NaCl, no sprayed AsA and LYC; NaCl, added 100 mmol/L NaCl; NA, added 100 mmol/L NaCl and sprayed 0.5 mmol/L AsA; NL, added 100 mmol/L NaCl and sprayed 0.25 mmol/L LYC; NLA, added 100 mmol/L NaCl and sprayed 0.25 mmol/L LYC and 0.5 mmol/L AsA.

In order to visualize and understand more deeply how exogenous AsA can alleviate the changes of photosynthetic apparatus caused by salt stress, based on the data in Figures 8, 9 (Supplementary Materials), the energy pipeline models of specific fluxes per RC and the phenomenological fluxes per excited cross-section (CS_m_) in the membrane of the tomatoes (Figure 10, left) and the leaf (Figure 10, right) under different treatments were constructed, respectively. Compared with the control, *F*_v_/*F*_m_ and *PI*_ABS_ values reduced significantly under salt stress (Figure 11). Applying the exogenous AsA significantly raises both the *PI*_ABS_ and *F*_v_/*F*_m_ values found in seedling leaves subjected to salt stress *(P* < 0.05). On the contrary, applying the exogenous LYC significantly decreases *F*_v_/*F*_m_ by 5.0% and *PI*_ABS_ by 10.6% in the salt-stressed leaves. NLA treatment significantly increased *F*_v_/*F*_m_ and *PI*_ABS_ compared with the NL treatment.

#### Photosystem I

**Figure 12:**
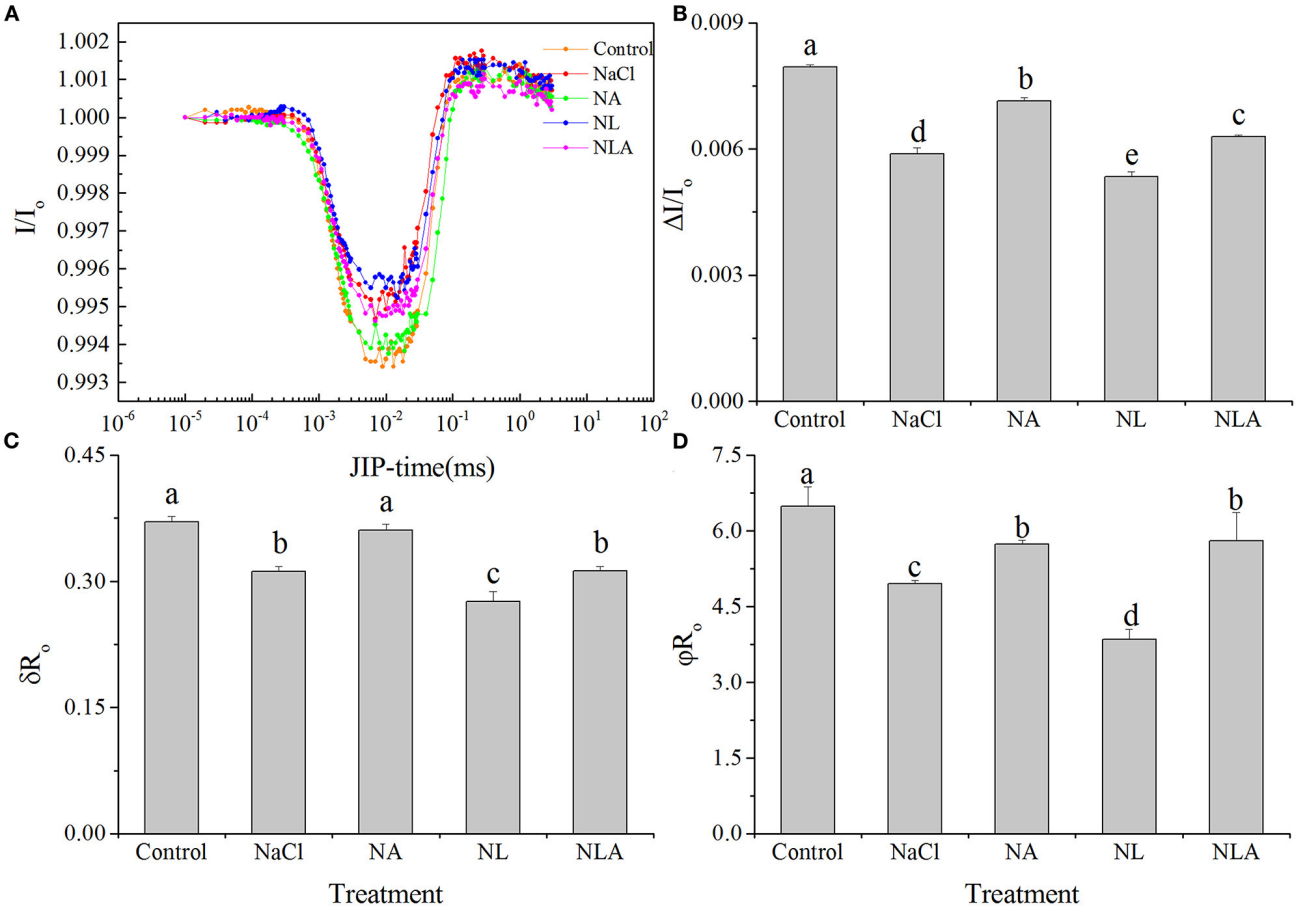
Curves of light induced modulated 820 nm reflection (*I*/*I*_o_) kinetics **(A)** and maximal redox capacity of PSI (Δ*I*/*I*_o_) **(B)**, efficiency with which an electron from the intersystem electron carriers is transferred to reduce end electron acceptors at the PSI acceptor side (δ*R*_o_) **(C)**, and quantum yield for reduction of end electron acceptors at the PSI acceptor side (ϕ*R*_o_) (D) in the leaves of salt-stressed tomato seedling with or without exogenous ascorbic acid (AsA) and lycorine (LYC) spraying. Values are means ± SD (*n* = 3). Different letters (a, b, c, d, and e) indicate significant difference at *P* < 0.05 among treatments. Error bar is smaller than the line at the top of the graph. Control, no added NaCl, no sprayed AsA and LYC; NaCl, added 100 mmol/L NaCl; NA, added 100 mmol/L NaCl and sprayed 0.5 mmol/L AsA; NL, added 100 mmol/L NaCl and sprayed 0.25 mmol/L LYC; NLA, added 100 mmol/L NaCl and sprayed 0.25 mmol/L LYC and 0.5 mmol/LAsA.

Δ*I*/*I*_o_, ϕ*R*_o_, and δ*R*_o_ are significantly and respectively decreased by 26.3%, 23.6%, and 15.8% (*P* < 0.05) in NaCl-stressed plants, compared with the plants used as controls (Figure 12). Applying AsA significantly raises δ*R*_o_, Δ*I*/*I*_o_, and ϕ*R*_o_ in the seedling leaves subjected to NaCl stress. In contrast, the application of LYC significantly decreases Δ*I*/*I*_o_, ϕ*R*_o_, and δ*R*_o_ in the plant leaves stressed by NaCl. Δ*I*/*I*_o_, ϕ*R*_o_, and δ*R*_o_ are significantly higher in the NLA treatment than that in the NL treatment.

## Discussion

Growth is the comprehensive embodiment of plant response to stress, and it is also a common index to determine stress tolerance (Liu et al., 2018). Salt stress is one of the primary abiotic factors affecting the quality and yield of crops (Landi et al., 2017). In this study, the results showed that AsA addition can alleviate the deleterious effects of salt stress (Figure 1; Table 2). This observation on the role of AsA in circumventing the adverse effects of salinity on plant biomass coincides with earlier reports (Wang et al., 2014; Terzi et al., 2015; Aliniaeifard et al., 2016). Meanwhile, we found that exogenous AsA application on salt-stress tomato seedlings is accompanied by an increase of endogenous AsA level and AsA/DHA ratio (Figure 2). Significant evidence demonstrates that the intracellular redox condition and the ROS scavenging capacity affect the resistance of abiotic stress in plants (Dietz, 2010). Keeping high redox pools and antioxidative level of GSH and AsA (or GSH/GSSG and AsA/DHA ratios) is necessary for plants to consume surplus ROS and maintain the sulfhydryl groups of membrane and soluble proteins in a lowered condition (Wang et al., 2020a). Our findings demonstrate that the exogenous application of AsA can induce tomato seedling growth under salt-stress conditions, and it was associated with increases in endogenous AsA levels. To further probe the role of AsA, we applied lycorine (LYC, an inhibitor of AsA synthesis key enzyme l-galactono-γ-lactone dehydrogenase activity) on the leaves of salt-stressed tomato seedlings prior or not to AsA treatment. LYC was used to reduce endogenous AsA content (Arrigoni et al., 1997). In this study, we observed that application of exogenous LYC under salt stress exacerbated growth inhibition and also significantly decreased the endogenous AsA level and the ratio of AsA/DHA (Figure 1; Table 2). However, the application of exogenous AsA can also effectively reverse the effects of LYC on the growth of tomato seedlings and the redox state of endogenous AsA (Figures 1, 2). These results give strong evidence that AsA can enhance the resistance of tomato seedlings to salt stress or salt stress with LYC application by mediating high reducing power and ROS scavenging capacity of endogenous AsA and redox state. This is consistent with the reports in barley by Agami (2014).

Under stressful environment, the decline in plant growth often relates to a decrease in photosynthetic capacity (Li et al., 2017; Zahoor et al., 2017). Reduced photosynthesis under salinity is not only attributed to stomatal closure factors but also attributed to non-stomatal limitation factors (Melesse and Caesar, 2010). Our findings demonstrate that a decrease in leaf *P*_n_ due to salt (Figure 3A) was mainly induced by non-stomatal factors, since salt-stress response elevated *C*_i_ (Figure 3D), even though *G*_s_ decreased (Figure 3B). In this experiment, exogenously applying AsA effectively combatted the negative effects of salt stress and NL treatment on *C*_i_, *G*_s_, and *P*_n_ in tomato seedling leaves (Figure 3). These results showed that AsA might help to relieve salt-induced photosynthetic inhibition mainly by the regulation of non-stomatal limitation factors, such as the decrease in photosynthetic pigments content, the chloroplast ultrastructure disorganization, the photochemical reaction activity decrease, or the decrease of enzyme activity involved in the process of carbon assimilation.

Chlorophyll molecules have high sensitivity to environmental stresses, and chlorophyll content, due to positive correlation with photosynthesis, is widely used as an index of abiotic tolerance in plants (An et al., 2018; Wang et al., 2020b). High salinity causes an increased ROS generation in chloroplasts, which can destroy chlorophyll. Also, reduction of chlorophyll content induced by salinity stress is due to the enhanced activity of chlorophyll enzymes and the instability of pigment–protein complex (Aghaleh et al., 2009; Hamid and Ashraf, 2010). In addition, salt stress reduces chlorophyll content by decreasing the uptake and accumulation of key nutrients such as Mg^2+^, Fe^2+^, and Cu^2+^, which are involved in the biosynthesis of chlorophyll (Evelin et al., 2012). The activities of Mg-chelatase and ferrochelatase are regulated by the redox state in chloroplast. Under the oxidized state in chloroplast, the activity of Mg-chelatase is inhibited and the activity of ferrochelatase is improved, finally resulting in the inhibition of chlorophyll biosynthesis (Walker and Willows, 1997; Adhikari et al., 2011). In this study, the reduced chlorophyll contents in salt-stressed plant leaves might be due to chlorophyll degradation and chlorophyll biosynthesis inhibition (Table 3). This is in line with what has been reported earlier in *Phaseolus vulgaris* L. (Taïbi et al., 2016), *Gossypium* spp (Saleh, 2012), and *Brassica chinensis* L. (Muhammad and Shafiq, 2014). AsA levels in plants increase when subjected to stress and serve important roles when defending against oxidative stress (Xu et al., 2015). Exogenous application of AsA has been reported to increase the chlorophyll contents in diverse plants (Saeidi-Sar et al., 2012; Ahmad et al., 2013; Kostopoulou et al., 2014). In this study, the exogenous application of AsA counteracted the adverse effects on photosynthetic pigments of salt stress with or without LYC (Figure 3; Table 3), and the AsA application to NaCl-treated or NL-treated tomato seedlings caused a considerable increase in the endogenous AsA content and AsA/DHA ratio (Figure 2), thereby promoting the biosynthesis of chlorophyll and protecting the chloroplasts from oxidative damage. Furthermore, higher carotenoids contents were also observed in the NA-treated and NLA-treated plants compared with the NaCl-treated and NL-treated plants (Table 3). Carotenoids can protect the photosynthetic apparatus from photooxidative damage by quenching the triplet state of chlorophyll and helping dissipate the excessive energy of excitation, thereby preventing the formation of ROS (Derks et al., 2015). Above all, these results suggest that alleviating chloroplasts oxidative stress damage and promoting chlorophyll synthesis, by enhancing the antioxidant capacity and modulating redox state in chloroplast of tomato seedlings under salt stress, is one of the mechanisms by which exogenous AsA could increase the photosynthetic capacity of salt-stressed tomato seedlings.

In order to further decipher the mechanism of action of salt stress on the electron transport chain and the mechanism of how exogenous AsA improve the performance of photosynthetic organs in salt-stressed tomato seedlings, the effects of AsA application on the structure and functioning of the photochemical apparatus of tomato seedlings under salt stress with or without LYC were investigated by rapid chlorophyll fluorescence technique. Our findings indicate that OJIP curve shapes are sensitive to salt stress. The OJIP curve was flatter in salt-stressed leaves than that in the control, because of decreases in *F*_m_ (P-step) and increases in *F*_o_ (O-step) (Figures 4A, 7). The normalized curve demonstrated relative J-step elevations, which can also be seen in the elevated V_J_ parameter, compared with that of control (Figure 7). These alterations in the values of *F*_o_, *F*_m_, and *V*_J_ suggest that the PSII donor end deteriorates as a result of salt stress and that the capacity of the PSII donor end to give electrons also decreases due to an increase in the closed PSII RCs (Strasser et al., 2010). The appearance of K- and L-bands in the OJIP transient curve of salt-treated leaves, as well as the increases in *W*_K_ and *W*_L_ values (Figures 5 and 6), also confirms the destruction of OEC and the integrity of the thylakoid membrane in the donor side of PSII, leading to the decrease in the electron donation capacity of the PSII donor side (Gomes et al., 2012; Li et al., 2014), as found for barley and passion fruit under drought stress (Oukarroum et al., 2007). The parameters, such as *V*_J_, *M*_o_, *S*_m_, ϕ*E*_o_, and ψ_o_, mainly reflect the changes of the acceptor side of PSII. According to the higher *V*_J_ in salt-treated leaves (Figure 7), we concluded that the acceptor side of PSII also suffered damage from salt stress. Photoinhibitory damage on the PSII acceptor end of leaves that have been treated with salt is confirmed by our observations that the stress of salt decreases *S*_m_, ϕ*E*_o_ and ψ_o_ and increases *M*_o_ (Figure 7) (Alaka Srivastava and Strasser, 2003; Oukarroum et al., 2004). Additionally, our results demonstrate that salt stress lowered the maximal redox threshold of PSI (*I*/*I*_o_ and Δ*I*/*I*_o_), the quantum yield necessary to reduce the end electron acceptors on the PSI acceptor end (ϕ*R*_o_), and how efficiently an electron is moved from the intersystem electron carriers to reduce the end electron accepted on the PSI acceptor end (δ*R*_o_) (Figure 12). These results indicate that salt stress reduced the capability of the photosynthetic electron transport from the PSII donor side and reduced the PSI end acceptors, reducing the photosynthetic capacity of the leaves. These findings suggest that both donor and acceptor sides of PSII and PSI are the target sites under salinity stress in tomato seedlings. Those results are consistent with the conclusions suggested by Shamshiri and Fattahi (2016) in pistachio (*Pistacia vera*). However, the application of AsA on the leaves of the tomato seedlings salt-stressed with or without LYC effectively reversed these changes on the donor side and acceptor side of photosystem (including PSII and PSI). These findings confirm that exogenously applying AsA serves an important role during the movement of electrons from the PSII donor end to the PSI acceptor ends. AsA can promote the electron transport activity (ϕ*E*_o_ and ψ_o_), enhance the PQ pool size (*S*_m_), and lower the rate of reduction of Q_A_ to ${\text{Q}}_{\text{A}}^{-}$ (Mo) on the PSII acceptor end. As such, there are increases in both the electron transport flux per reaction center (ET_o_/RC) and the primary photochemical efficiency (ψ_o_), improving the efficiency of photochemical processes in tomato seedlings under salt stress.

Regulating the activity of PSII reaction centers and improving the ability of chloroplasts to absorb and dissipate energy is the key to improving plant salt tolerance. In this study, the increase of ABS/CS_m_, TR/CS_m_, and ET_o_/CS_m_ in tomato seedlings under salt stress (Figures 9, 10) may be a protective mechanism under salt stress (Sane et al., 2002). In contrast, the decreased ABS/RC and TR_o_/RC under salt stress (Figures 9, 10) means that the effective average absorption of the antenna and trapping per active RC are both higher because of certain RC inactivation (Yusuf et al., 2010). The increased DI_o_/RC indicates that the proportion of total dissipation to active RCs increases due to high dissipation of the inactive RCs (Gerosa et al., 2003), which is consistent with previous results in cucumber under salt stress (Yuan et al., 2014). However, the negative effects of salt stress (in the presence of LYC or not) on active RC density and the use and expenditure of absorbed energy in PSII RCs were counteracted by exogenous application of AsA. The majority of plants dissipate excitation energy surpluses thermally, along with decreased regulation of PSII activity. This defends the photosynthetic apparatus abiotic environmental stressors, which can damage them. Xu (2017) reported that exogenous AsA can effectively promote xanthophyll loops in wheat under Cr^6+^ stress to dissipate excessive excitation light energy, thus avoiding the occurrence of photoinhition. So, our results suggest that AsA may act as a cofactor of violaxanthin de-epoxidase in the xanthophyll cycle (Muller-Moule et al., 2002) which helps protects tomatoes against the harmful effects of excess excitation energy induced by salt stress. Additionally, the role of AsA in regulating the photosynthetic capacity has been extensively studied by assessing patterns in the fluorescence parameters *F*_v_/*F*_m_ as well as the performance index *PI*_ABS_. We determined that lower *F*_v/_*F*_m_ levels in the leaves of tomatoes due to salt stress were associated with lower levels of *PI*_ABS_ (Figure 11), suggesting that salt-induced photoinhibition was due to damaged photosynthetic apparatus caused by excess light energy absorption compared with the level required for photosynthesis and limited component mechanism efficiency like the movement of electrons within a system and the trapping of energy (Song et al., 2003; Campos et al., 2014). The results in Figure 11 show that AsA played a key role in regulating the use and expenditure of energy absorbed by PSII RCs in seedlings subjected to salt stress, both in the presence of LYC and without LYC. This is supported by the increased *F*_v_/*F*_m_ and *PI*_ABS_ values (Figure 11). Therefore, these findings indicate that photosynthetic defense due to AsA was a result of increased capacity not only to protect photosynthetic processes from the negative effects of salt stress but also to inactivate the RCs of PSII replacement and regulation of the light striking PSII RCs, which is where absorbed energy is captured and energy from the dissipation of heat is transferred to a photochemical reaction. Additional energy from excitation was converted to transport electrons, a process that could provide additional transport capacity for electrons on the PSII donor end. As such AsA serves an important role in protecting the photosynthetic capacity during salt-stress conditions.

## Conclusion

In conclusion, this study clearly demonstrated that salt stress lowered the capacity of several parts of the photosynthetic electron transport path. The OEC was damaged, PSII RCs were inactivated, the connections among independent PSII units were decreased, there was little electron transport beyond Q_A_, and electron transporters on the PSI acceptor end in the leaves of tomato seedlings were damaged. However, exogenously applying AsA decreased the photoinhibition and lessened the negative effects on photosynthesis. Thus, under salt-stress conditions AsA can enhance salt-stress tolerance by promoting chlorophyll synthesis and alleviate the damage of oxidative stress to chloroplasts by modulating the redox state in chloroplast and dissipating excitation energy in the PSII antennae. AsA can also stabilize the molecular structure of photosynthetic reaction center, keeping the electron transport chain opened and enhancing the electron transfer efficiency, which is helpful to promote the improvement of photochemical activity and photosynthetic performance and the efficient distribution of energy. Moreover, the several beneficial effects of AsA during salt stress make AsA a good candidate for potential use as a chemical in agriculture.

## Data Availability Statement

The original contributions presented in the study are included in the article/Supplementary Material, further inquiries can be directed to the corresponding author/s.

## Author Contributions

The work presented here was carried out in collaboration between all authors. H-yL, MD, and XC defined the research theme. XC designed methods and experiments, carried out the laboratory experiments, analyzed the data, interpreted the results, and wrote the manuscript. YZ co-designed the experiments, carried out the laboratory experiments, and discussed the analyses and interpretation. YC, PZ, JC, WX, and QS co-worked on the analysis of fast fluorescence induction kinetics curve and JIP test parameters and discussed analyses and interpretation. H-yL and MD conceived and coordinated the study. All authors have contributed to, seen, and approved the final manuscript.

## Conflict of Interest

The authors declare that the research was conducted in the absence of any commercial or financial relationships that could be construed as a potential conflict of interest.

## Publisher's Note

All claims expressed in this article are solely those of the authors and do not necessarily represent those of their affiliated organizations, or those of the publisher, the editors and the reviewers. Any product that may be evaluated in this article, or claim that may be made by its manufacturer, is not guaranteed or endorsed by the publisher.
